# Identification and expression profiling of LSD genes reveal their role in developmental and abiotic stress conditions in maize

**DOI:** 10.3389/fpls.2026.1760884

**Published:** 2026-02-10

**Authors:** Dongbo Zhao, Longxue Wei, Jianjun Guo, Zhihui Guo, Lianghai Guo, Jiansheng Gao, Huini Cui, Rongjian Tai, Peiyan Guan, Liang Zhang, Peng Liu, Yirong Jin

**Affiliations:** 1 Food Crops Research Institute, Dezhou Academy of Agricultural Science, Dezhou, Shandong, China; 2 College of Energy and Machinery, Dezhou University, Dezhou, Shandong, China; 3 College of Life Science, Dezhou University, Dezhou, Shandong, China

**Keywords:** abiotic stress response, expression patterns analysis, genome-wide analysis, hormone response, LSD gene family, maize

## Abstract

The Lesion Simulating Disease (LSD) genes encode a class of zinc finger proteins that play crucial roles in hypersensitive responses and programmed cell death (PCD) triggered by biotic and abiotic stresses. However, the comprehensive genome-wide identification of the LSD family in maize, comparative synteny analysis, and systematic tissue- and stress-specific expression profiling remain poorly understood. In this study, we systematically identified and characterized the LSD gene family at the genome-wide level in maize. Bioinformatics methods were employed to analyze the physical and chemical properties, chromosomal location, phylogenetic relationships, conserved motifs, and gene structure of the LSD gene family members. The expression patterns of the *ZmLSDs* under the conditions of drought, high temperature, high salt, and hormone treatment with ABA, were detected by RT-qPCR. The subcellular localization of the ZmLSDs was observed by laser confocal microscopy. A total of nine LSD genes encoding 23 protein isoforms was identified from the maize genome and named *ZmLSD1* to *ZmLSD9*. ZmLSD family proteins have 113–898 amino acids, relative molecular weights ranging from 12.133 to 93.568 KD. The ZmLSD gene family members were distributed on five chromosomes, mainly on Chr1 and Chr3. According to phylogenetic analysis, the ZmLSD family members can be divided into four subfamilies. Motif analysis revealed that Motif1 is the conserved motif shared by these genes, which is presumably related to the conserved structural domain. There were three intra-species covariance gene pairs, and seven *ZmLSDs* exhibited syntenic homologs with both sorghum and japonica rice LSD genes. *ZmLSD3*, *ZmLSD4*, and *ZmLSD9*, were expressed at higher levels in all tissue sites, except the embryo and endosperm. Expression profiling analysis showed that *ZmLSDs* can respond to drought, high temperature, high salt, and ABA hormone, especially most of the genes were down-regulated significantly after heat and drought stress treatments, which indicated that *ZmLSDs* play an important role in coping with abiotic stress in maize. ZmLSD3 was mainly distributed in the cytoplasm, while ZmLSD4 was distributed in both the nucleus and cytoplasm. The above results indicate that the LSD gene family plays an important role in regulating abiotic stress and hormone ABA responses during maize growth and development.

## 1 Introduction

The Lesion Simulating Disease (LSD) gene family was originally identified in the *Arabidopsis thaliana*, which functions as programmed cell death (PCD) negative regulators involved in plant disease resistance defense responses (Dietrich et al., 1994, 1997). Three LSD genes were identified in Arabidopsis, including *LSD1*, *LOL1* (LSD-one-like1) and *LOL2* (LSD-one-like 2) (Epple et al., 2003). They contain one to three specialized zinc finger structure (zf-LSD1) with the shared sequence CxxCRxxLMYxxGASxVxCxxC (Dietrich et al., 1997).

Further research has found that LSD family genes exhibit diverse functions in the growth and development of plants. They not only participate in regulating plant hypersensitive responses (HR) and transmitting disease resistance signals (Dietrich et al., 1994, 1997) but also modulate plant responses to both biotic and abiotic stresses (Cabreira et al., 2013; Guan et al., 2016; Jiang et al., 2019). LSD family genes can respond to *Phakopsora pachyrhizi* infection and dehydration in soybean (Cabreira et al., 2013). In poplar, *PagLSDs* can be induced by polyethylene glycol (PEG) or ABA, and overexpression of *PagLOL1b* significantly enhanced the drought tolerance of transgenic plants (Chao et al., 2024). Under excess light energy conditions, *AtLSD1* regulates stomatal closure, enhances ROS scavenging and prevents plant photo-damage (Mateo et al., 2004). During cold stress, *AtLSD1* inhibits cell death by regulating ROS responses (Jabs et al., 1996). However, *AtLOL1* acts as a positive regulator of PCD and regulates oxidative stress-induced cell death antagonistically with AtLSD1 proteins (Epple et al., 2003). LSD1 and MC1 may play a key role in AtLSD1 deathosome, exhibiting physical interactions with multiple protein families involved in Arabidopsis HR-PCD (Valandro et al., 2020). Later researchers cloned several LSD1-like genes in rice, bamboo, pepper, and other plants. In rice, *OsLOL1* not only acts as a negative regulator of PCD but also plays a positive role in healing tissue differentiation and promotion of seed germination by regulating gibberellin biosynthesis (Wang et al., 2005; Wu et al., 2014). Overexpression of *OsLOL5* in rice and Arabidopsis enhances plant tolerance to alkaline stress (Guan et al., 2016). Overexpressing *ZmLSD1* in maize obviously enhanced the tolerance of plants to salt stress (Li et al., 2024). In bamboo, *BohLOL1* is involved in bamboo growth and response to biotic and abiotic stress, which expression was changed after heat-stress and H_2_O_2_ treatments (Yeh et al., 2011). *CcLOLl* can regulate chloroplast compartment size and chlorophyll content in pepper (Borovsky et al., 2019). In addition, in *Rosa hybrida*, RhLOL1-RhILR3 regulatory module participates in the cytokinin-induced petal abscission process by regulating of the expression of the Aux/IAA genes (Jiang et al., 2023). *LSD1* can regulate salicylic acid accumulation in *Arabidopsis thaliana*, enhancing the plant’s resistance to UV A+B stress (Bernacki et al., 2021). Therefore, LSD gene family plays a crucial role in plants’ responses to abiotic stress and hormonal processes.

Maize is the most important food crop in China, with large production potential and high economic benefits. However, during its growth and development, abiotic stress (such as drought, heat, salinity, etc.) can seriously affect the yield and quality of maize. Therefore, it is of great significance to explore the functions of stress-related genes and decipher their regulatory mechanisms to improve maize resistance. Although some LSD genes (such as *ZmLSD1*) have been reported to play an important role in biotic and abiotic stresses, the comprehensive genome-wide identification of the LSD family in maize, comparative synteny analysis, and systematic tissue- and stress-specific expression profiling remain unclear. In this study, we conducted a comprehensive analysis of the phylogenetic relationship, gene structure, conserved motifs, *cis*-elements, and chromosomal distribution of maize LSD genes, and investigate their expression patterns under abiotic stress, to provide theoretical support for the further exploration of the functions and regulatory mechanisms of maize LSD genes.

## 2 Materials and methods

### 2.1 Plant materials and treatments

Seeds of maize inbred line B73 were cultivated in a growth chamber under conditions of 16h light, 25 °C, and 8h darkness, 22 °C. The seedlings at the three-leaf stage were subjected to the corresponding stress treatments (Guan et al., 2025). For drought treatment, the seedlings were watered with 35% PEG-6000 (w/v) for 0, 1, 3, 6, 12, and 24 h, respectively. For high salt treatment, the seedlings were watered with 200 mM NaCl solution. Samples were collected at 0, 1, 3, 6, 12 and 24 h after the treatment. Seedlings were subjected to heat stress treatment in a plant growth chamber maintained at 42 °C for 0, 4, and 8 h, respectively. For hormone treatment, seedlings were sprayed with 100 μM abscisic acid (ABA), and collected at various time intervals (0, 1, 3, 6, 12, and 24 h). Seedlings with no treatment (0 h) served as control. Leaf samples were harvested at the designated time points and snap-frozen in liquid nitrogen, then stored at -80 °C for subsequent RNA extraction.

### 2.2 Identification of LSD family members in maize genome

The genomes fasta file, gff3 file, and protein fasta file of four species, including *Zea mays* (Zm-B73-REFERENCE-NAM-5.0.55), *Arabidopsis thaliana* (TAIR10), *Sorghum bicolor* (NCBIv3), and *Oryza sativa* subsp. japonica (IRGSP), were downloaded from Phytozome database (https://phytozome-next.jgi.doe.gov) (Goodstein et al., 2012) (accessed on 10 March 2025). The structural domain file Pfam-A.hmm was downloaded from the Pfam database (http://pfam.xfam.org/) (Mistry et al., 2021) (accessed on 11 March 2025). The Simple HMM Search function in the TBtools V2.110 (Chen et al., 2023) software was used to identify the maize LSD family, by using all the protein sequences of maize and the structural domain login number of the LSD family (PF06943.16, LSD1 zinc finger), and the E-value was set to 10^-5^. The Domain of the protein obtained was analyzed by using the online software NCBI Batch CD-Search (https://www.ncbi.nlm.nih.gov/Structure/bwrpsb/bwrpsb.cgi) (Wang et al., 2023) (accessed on 12 March 2025). The common sequence of the zinc finger structural domain of the LSD1 gene family was checked by aligning the sequences with MEGA V11.0 (Mega Limited, Auckland, New Zealand) (Tamura et al., 2021).

### 2.3 Analysis of physicochemical properties of ZmLSD family members proteins

Physicochemical characteristics such as the number of amino acids, molecular weight, theoretical isoelectric point, instability index, etc. of ZmLSD family members were analyzed by using the online tool Expasy ProtParam (http://web.expasy.org/protparam/) (Wilkins et al., 1999). The subcellular localization of ZmLSD family members was predicted using the online tool WoLF PSORT (https://wolfpsort.hgc.jp/) (Horton et al., 2007).

### 2.4 Evolutionary analysis of the ZmLSD family

The protein sequences of the LSD family members of Arabidopsis, sorghum, and japonica rice were downloaded from PlantTFDB (https://planttfdb.gao-lab.org/index.php?sp=Zma) (Jin et al., 2017). Using the software MEGA V11.0, the phylogenetic tree of the LSD families in these four species was constructed using the neighbor-joining (NJ) method, with bootstrap replicates set to 1000 and other parameters as system defaults (Tamura et al., 2021). The phylogenetic tree was display using the iTOL V6 (https://itol.embl.de/) (Letunic and Bork, 2024).

### 2.5 Gene structure and protein conserved motif analysis of ZmLSD family members

Based on the genome fasta and gff3 annotation file, the CDS sequences were obtained. According to the genome sequence and CDS sequence, the gene structure of ZmLSD family members can be obtained by using TBtools V2.110 software. The protein conserved motifs of the maize LSD gene family members were analyzed using the online tool MEME (https://meme-suite.org/meme/) (Bailey et al., 2015), and the maximum number of Motifs was set to 3. The Domain of LSD genes was analyzed by using SMART (http://smart.embl-heidelberg.de/) (Letunic et al., 2021), and visual mapping was performed using TBtools V2.110.

### 2.6 Chromosomal localization and covariance analysis of ZmLSD family members

From the maize whole genome annotation file (gff3), the position information of ZmLSD family genes on chromosomes was extracted. The chromosome location maps were drawn based on the location of the genes on the chromosomes. The covariance relationship within the maize genome was analyzed using the MCScanX toolkit in the TBtools V2.110 (Wang et al., 2012).

### 2.7 Analysis of *cis*-acting elements on promoters of ZmLSD family members

Based on the gff3 annotation file and genome sequences, the CDS and promoter (2000 base pairs (bp) upstream of the start codon ATG) sequences of *ZmLSDs* were extracted using TBtools V2.110. *Cis*-acting elements on each *ZmLSD* promoter was predicted using PlantCARE (http://bioinformatics.psb.ugent.be/webtools/plantcare/html/) (Lescot et al., 2002).

### 2.8 Analysis of ZmLSD gene expression patterns in tissues and under abiotic stress

Transcriptome data of *ZmLSD* gene expression patterns were obtained from the NCBI database accession numbers PRJNA171684 and SRP010680 (Stelpflug et al., 2016), and the heat map was drawn using TBtools software V2.110.

Total RNA was extracted using RNAiso Plus (TaKaRa, Beijing, Japan). After testing for purity and quality using Nanodrop 2000 spectrophotometer (Thermo Scientific), 1 μg of total RNA was reverse-transcribed into first-strand cDNA using Evo M-MLV RT Kit with gDNA Clean for qPCR (Accurate Biology, Hunan) according to the manufacturer’s instructions. The cDNA template was diluted for 30-fold. The qRT-PCR reaction system consisted of 7.5 μL of SYBR Green *Pro Taq* HS Premix (Accurate Biology, Hunan), 0.3 μL upstream and downstream specific primers, 1.9 μL of ddH_2_O and 5 μL template. The reaction program was pre-denaturation at 95 °C for 30 sec; denaturation at 95 °C for 5 sec, annealing and extension at 60 °C for 30 sec, and the samples underwent 45 amplification cycles. After completion, a melting curve was recorded by setting the temperature to start at 65 °C and gradually increased at 0.5 °C/s until 95 °C. Three biological replicates and three technical replicates per target gene were performed for qPCR. Ct values from technical replicates were averaged to reduce noise. *ZmActin 1* was regarded as reference gene and the relative expression levels of *ZmLSDs* were calculated using the 2^-△△Ct^ method (Schmittgen and Livak, 2008). The primer sequences used were listed in **Supplementary Table 1**.

### 2.9 Statistical analysis

All the experimental measurements were repeated for three times. Data processing was performed with Microsoft Excel 2021, while statistical plotting, analysis of variance, and comparisons of differences (Student’s *t*-test) were completed with GraphPad Prism 6 (GraphPad Software Inc.; San Diego, CA, USA). Significant difference was defined as *p* < 0.05 (*) and *p* < 0.01 (**).

## 3 Results

### 3.1 Identification and characterization of *ZmLSDs* in maize

For analyzing the LSD gene family in maize, the Simple HMM Search toolkit in the TBtools wasemployed to search LSD genes against local maize genome databases. After NCBI’s Conserved Domain Database (CDD) verification, a total of 23 proteins containing conserved structural domains of the LSD were identified at the genome-wide level, encoded by nine genes location (**Supplementary Table 2**). The genes were renamed *ZmLSD1* to *ZmLSD9* based on their location on the chromosome, and 23 protein isoforms were renamed ZmLSD1.1 to ZmLSD9.3 (**Table 1**). The number of amino acids of the ZmLSD family members varied from 113 to 898, the relative molecular weights ranged from 12.133 KD to 93.568 KD, and the theoretical isoelectric points (pI) ranged from 4.46 to 9.63. Among them, ZmLSD7.2 protein sequence has the shortest length and the smallest molecular weight, which is only 12133.08 Da. There were 14 basic proteins (pI values greater than pH 7.0) and 9 acidic proteins (pI values less than pH 7.0). The instability index of ZmLSD ranged from 33.77 to 77.39, with 9 proteins with instability index less than 40 and 14 proteins with index greater than 40, indicating most ZmLSD protein structures were unstable. The values of grand average of hydropathicity (GRAVY) indicated most proteins were hydrophobic proteins. Subcellular localization prediction results showed that four members were localized in the nucleus, one (ZmLSD3.3) was located in the cytoplasm, and eighteen were localized in the chloroplasts (**Table 1**). Secondary structure analysis revealed that all ZmLSD proteins contain alpha helix,extended strand, beta turn, and random coil. ZmLSD1 and ZmLSD9 exhibit relatively high proportions of alpha helix and extended strand, whereas ZmLSD5 contains over 90% random coil (**Supplementary Table 2**). These results demonstrate significant divergence in the basic properties of ZmLSDs, implying functional diversity among family members.

**Table 1:** Basic information of ZmLSD family members.

Gene ID	Protein name	Protein ID	Number of amino acid	Molecular weight	Theoretical pI	Instability index	Aliphatic index	GRAVY	Subcellular localization
*ZmLSD1*	Zm00001eb018930_P001	ZmLSD1.1	423	45665.02	9.1	47.2	79.62	-0.147	Chloroplast
	Zm00001eb018930_P002	ZmLSD1.2	378	40641.31	8.52	39.92	79.81	-0.148	Chloroplast
*ZmLSD2*	Zm00001eb023930_P001	ZmLSD2.1	351	38050.27	5.98	39.97	72.25	-0.244	Chloroplast
	Zm00001eb023930_P002	ZmLSD2.2	351	38050.27	5.98	39.97	72.25	-0.244	Chloroplast
*ZmLSD3*	Zm00001eb050760_P001	ZmLSD3.1	175	18288.11	8.68	33.77	77.31	0.116	Chloroplast
	Zm00001eb050760_P002	ZmLSD3.2	175	18288.11	8.68	33.77	77.31	0.116	Chloroplast
	Zm00001eb050760_P003	ZmLSD3.3	175	18288.11	8.68	33.77	77.31	0.116	Cytoplasm
*ZmLSD4*	Zm00001eb135920_P001	ZmLSD4.1	169	17880.18	8.53	36.85	89.82	0.446	Chloroplast
	Zm00001eb135920_P002	ZmLSD4.2	178	18659.01	8.8	34.76	89.61	0.393	Chloroplast
	Zm00001eb135920_P003	ZmLSD4.3	309	33165.91	9.06	40.55	86.67	0.278	Chloroplast
	Zm00001eb135920_P004	ZmLSD4.4	178	18659.01	8.8	34.76	89.61	0.393	Nucleus
	Zm00001eb135920_P005	ZmLSD4.5	201	21494.33	9.24	43.95	85.17	0.248	Nucleus
*ZmLSD5*	Zm00001eb161950_P001	ZmLSD5.1	885	92594.42	4.52	75.19	74.68	-0.27	Nucleus
	Zm00001eb161950_P002	ZmLSD5.2	824	86104.02	4.52	77.39	73.23	-0.305	Chloroplast
	Zm00001eb161950_P003	ZmLSD5.3	860	89939.57	4.54	75.86	74.8	-0.26	Chloroplast
	Zm00001eb161950_P005	ZmLSD5.4	898	93568.51	4.46	73.47	75.12	-0.235	Nucleus
*ZmLSD6*	Zm00001eb172290_P001	ZmLSD6.1	198	21531.2	8.84	44.45	74.34	0.168	Chloroplast
*ZmLSD7*	Zm00001eb173250_P001	ZmLSD7.1	115	12216.16	9.45	41.34	66	-0.209	Chloroplast
	Zm00001eb173250_P002	ZmLSD7.2	113	12133.08	9.63	40.19	65.4	-0.247	Chloroplast
*ZmLSD8*	Zm00001eb261760_P001	ZmLSD8.1	150	15809.16	9.42	45.43	63.73	-0.235	Chloroplast
*ZmLSD9*	Zm00001eb390830_P001	ZmLSD9.1	351	38007.26	6.16	44.23	73.13	-0.227	Chloroplast
	Zm00001eb390830_P002	ZmLSD9.2	351	38007.26	6.16	44.23	73.13	-0.227	Chloroplast
	Zm00001eb390830_P003	ZmLSD9.3	351	38007.26	6.16	44.23	73.13	-0.227	Chloroplast

### 3.2 Evolutionary analysis of ZmLSD family members

**Figure 1:**
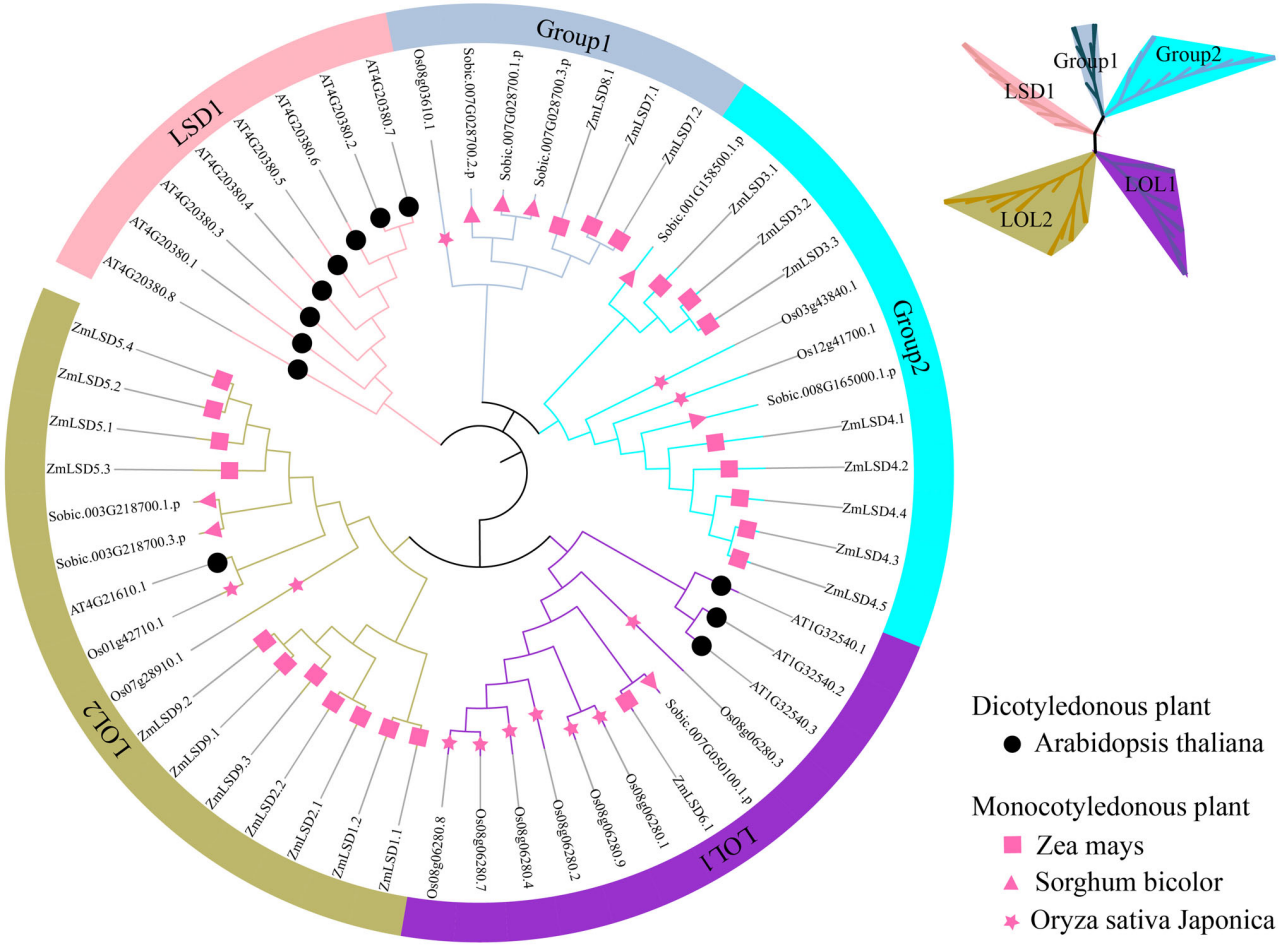
Phylogenetic analysis of LSD gene family proteins in *Zea mays*, *Arabidopsis thaliana*, *Sorghum bicolor*, and *Oryza sativa* Japonica. The phylogenetic tree was constructed using MEGA software based on protein sequences by the neighbor-joining method with 1000 bootstrap replicates. Black symbols denote dicotyledonous plants, while red symbols correspond to monocotyledonous plants. The red square indicates *Zea mays* LSDs (ZmLSD), red star indicates *Oryza sativa* Japonica LSDs, red triangle indicates *Sorghum bicolor* LSDs, and black circle indicates *Arabidopsis thaliana* LSDs. The 55 LSDs can be divided into five subfamilies: LSD1, Group 1, Group 2, LOL1, and LOL2. The Arabidopsis LSD family contains AtLSD1, AtLOL1 and AtLOL2, which correspond to three subfamilies: LSD1, LOL1 and LOL2, respectively. The two subclasses that do not contain Arabidopsis LSD family members are named Group 1 and Group 2.

The MEGA software was used to construct a phylogenetic tree containing 55 LSD proteins, including 12 proteins from Arabidopsis, 8 proteins from sorghum, 12 proteins from japonica rice and 23 proteins from maize. The phylogenetic tree was annotated with the online software iTOL. According to the evolutionary relationships, the 55 LSD members can be divided into five subfamilies: LSD1, Group 1, Group 2, LOL1, and LOL2 (**Figure 1**). In LSD1, there are only Arabidopsis members belonging to dicotyledonous plants, while maize, sorghum and japonica members belonging to monocotyledonous plants are absent. Group 1 contains three ZmLSDs and group 2 has eight ZmLSDs, while LOL1 contains only one ZmLSD member, namely ZmLSD6.1. The largest number of ZmLSD family members belong to LOL2, with eleven members. The results indicate that LSD1, Group 1, and Group 2 have remained highly conserved throughout plant evolution, whilst LOL1 and the LOL2 family underwent expansion during the evolution of monocotyledons and dicotyledons.

### 3.3 Gene structure, motif and domain analysis of ZmLSD family members

**Figure 2:**
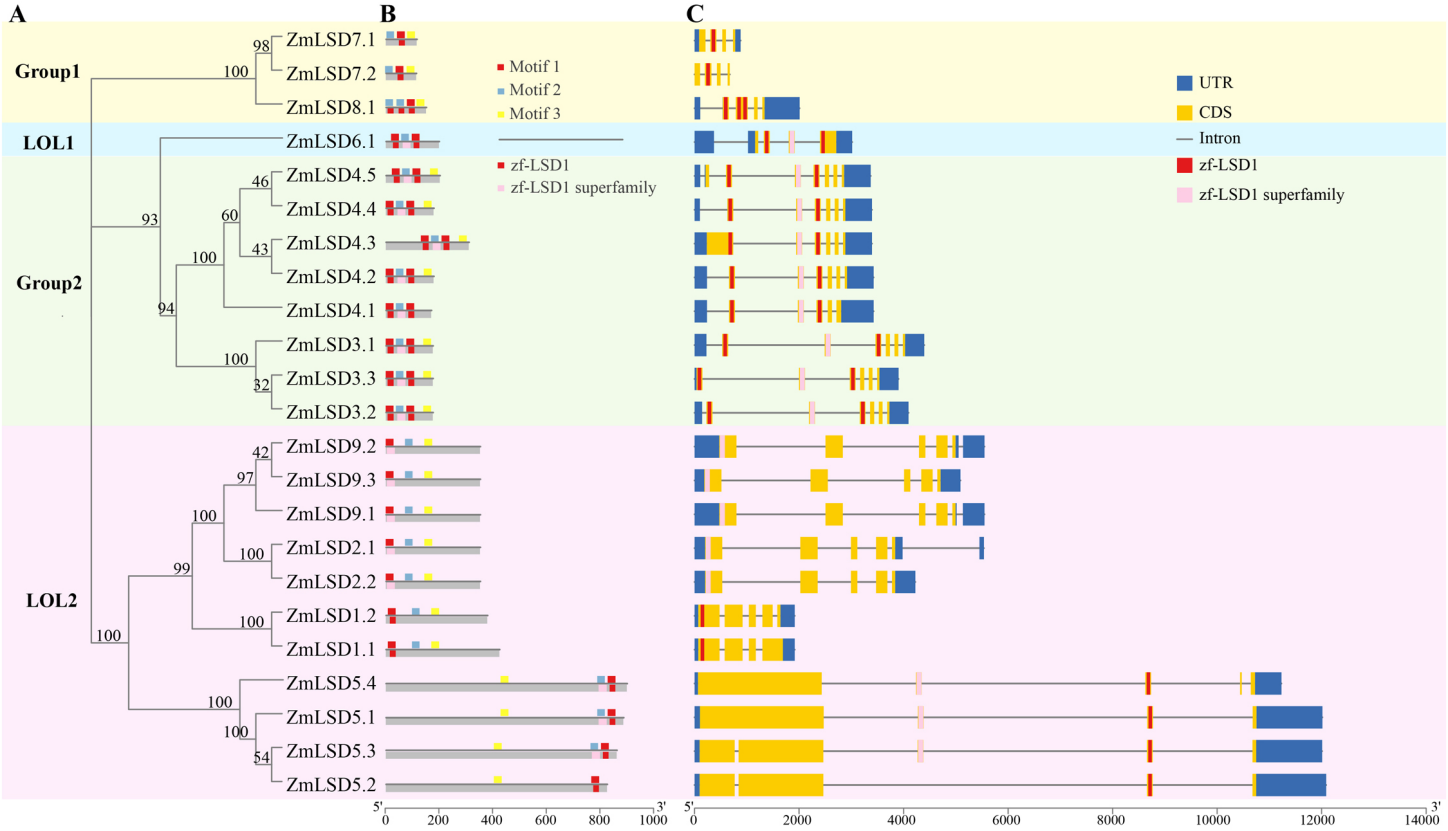
Phylogenetic tree, conserve motifs and domains, and gene structure of ZmLSD family members. **(A)** Phylogenetic tree of ZmLSD family members. **(B)** Corresponding diagram of conserved motifs and domain positions in ZmLSD family members, with Motif distribution positions labeled above the gray line and Domain distribution positions labeled below the gray line. **(C)** Gene structure diagram of ZmLSD family members, primarily showing UTR, CDS, and Intron regions, with the CDS containing the gene structure corresponding to the domains.

We constructed a phylogenetic tree using the protein sequences of 23 maize LSD family proteins. The results showed that ZmLSD protein classification was consistent with **Figure 1** (**Figure 2A**). In order to investigate the protein conservation of ZmLSD family members, MEME online software was used to predict the conserved Motifs of proteins with a maximum of 3 Motifs. Meanwhile, the online software Conserved Domain Database in NCBI was used to predict the domain of ZmLSD family members. The results showed that 23 ZmLSDs all contained Motif1, and there was a correspondence between Motif1 and zf-LSD1 or zf-LSD1 superfamily in position (**Figure 2B**). Motif1 contained the C2C2 zinc finger structure, and the conserved domain of its zinc finger structure consisted of 22 amino acids with the sequence CxxCxxxLxxxxGAxxxxCxxC (**Supplementary Figure 1**).

Analysis of the gene structure of ZmLSD family members revealed that ZmLSD family members consist of 4–7 exons. The largest number of members contained five exons, including five genes (nine transcripts), namely *ZmLSD4.1*, *ZmLSD9.2*, *ZmLSD9.3*, *ZmLSD9.1*, *ZmLSD2.1*, *ZmLSD2.2*, *ZmLSD1.2*, *ZmLSD5.4*, *ZmLSD5.3*; followed by genes containing four exons, consisting of seven transcripts (*ZmLSD7.1*, *ZmLSD7.2*, *ZmLSD8.1*, *ZmLSD6.1*, *ZmLSD1.1*, *ZmLSD5.1*, *ZmLSD5.2*). There were six transcripts containing six exons: *ZmLSD4.4*, *ZmLSD4.3*, *ZmLSD4.2*, *ZmLSD3.1*, *ZmLSD3.3*, *ZmLSD3.2*; One gene containing seven exons (*ZmLSD4.5*). The *ZmLSD7.2* did not have UTR, while the remaining 22 ZmLSD members all contained 5’ and 3’UTRs. The 5’ UTR of *ZmLSD6.1* is spaced by an intron, and the 3’ UTR of *ZmLSD9.1*, *ZmLSD9.2*, and *ZmLSD2.1*, are spaced by an intron. The corresponding positions of domains were made in the gene structure, and it was found that Zf-LSD1 and Zf-LSD1 superfamily were mostly distributed on different exon fragments (**Figure 2C**).

### 3.4 Chromosomal localization and covariance analysis of ZmLSD gene family members

**Figure 3:**
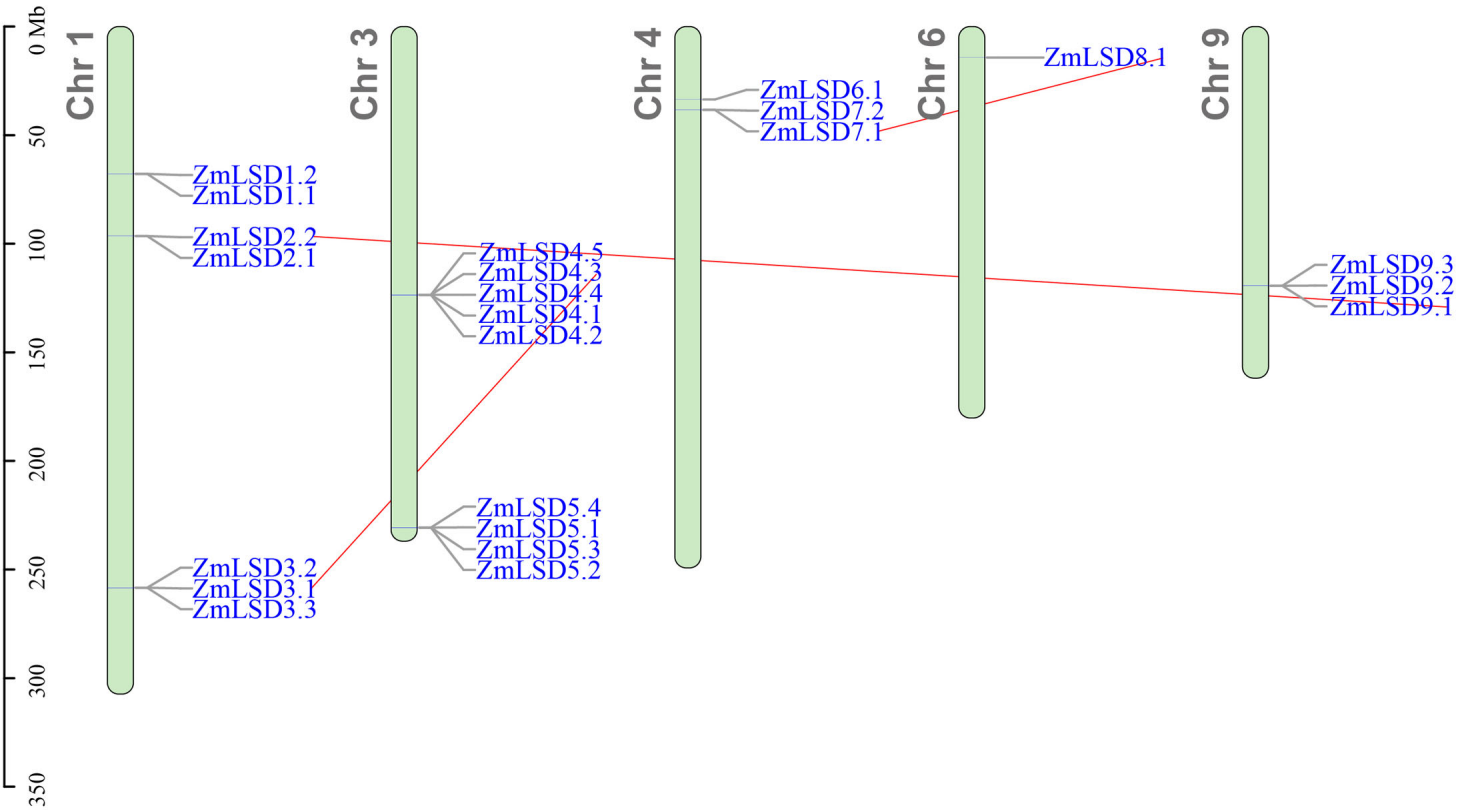
The location of ZmLSD family members on chromosome. Chromosome numbers are on the left and ZmLSDs are on the right of chromosomes. Scale bar on the left indicates chromosome length. Red line connects collinear gene pairs.

To clearly illustrate the abundance and chromosomal distribution patterns of ZmLSD gene family members, we employed TBtools software for visualization. As demonstrated in **Figure 3**, nine genes exhibit a non-random distribution across five maize chromosomes, with notable clustering observed on specific chromosomal regions. They are mainly distributed on Chr1 and Chr3, with three genes (corresponding to seven transcripts) and two genes (corresponding to nine transcripts), respectively. Chr4 has two genes (three transcripts), Chr6 has one gene (one transcript), and Chr9 has one gene (three transcripts).

The collinear relationships of ZmLSD family members within the maize genome and among other species, including *Arabidopsis*, sorghum, and rice were analyzed respectively using the MCScanX function in the TBtools software. There were three intra-species covariance gene pairs for the ZmLSD family members, namely, ZmLSD2.2 and ZmLSD9.1, ZmLSD3.1 and ZmLSD4.3, and ZmLSD7.1 and ZmLSD8.1 (**Figure 3**). The ZmLSD1.1 and Zm00001eb392460_T002 are also a covariant gene pair, butZm00001eb392460_T002 does not belong to the ZmLSD family (**Supplementary Table 3**).

**Figure 4:**
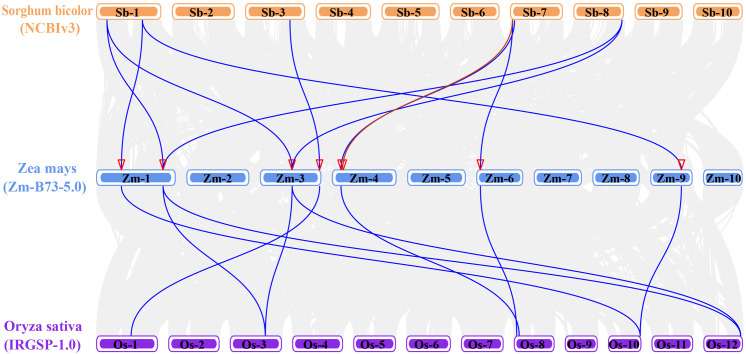
Syntenic gene pairs of LSD genes among *Zea mays*, *Sorghum bicolor*, and *Oryza sativa* subsp. japonica. Colored rectangles represent chromosomal segments. Blue denotes *Zea mays*, orange denotes *Sorghum bicolor*, and purple denotes *Oryza sativa* subsp. japonica. Syntenic gene pairs are depicted with blue arcs, while red arcs specifically highlight syntenic gene pairs to the sorghum-maize lineage. Red triangles indicate the location of ZmLSD family members on chromosome.

Comparative genomics analyses reveals that maize LSD genes exhibited a significant divergence in syntenic relationships between monocot and dicot species. No syntenic gene pairs were detected between the ZmLSD family and dicotyledonous plants, including *Arabidopsis thaliana*, soybean (*Glycine max*), or cabbage (*Brassica oleracea*), indicating lineage-specific genomic reorganization after species divergence. Strikingly, synteny was observed with monocot species, particularly sorghum (*Sorghum bicolor*) and rice (*Oryza sativa subsp*. japonica). There are seven *ZmLSD* transcripts, namely *ZmLSD2.2*, *ZmLSD3.1*, *ZmLSD4.3*, *ZmLSD5.4*, *ZmLSD6.1*, *ZmLSD8.1*, *ZmLSD9.1*, exhibited syntenic homologs with both sorghum and japonica rice LSD genes (**Figure 4**). However, *ZmLSD7.1* exhibits a unique colinear relationship only with the sorghum LSD gene, not with the rice LSD gene (**Figure 4**, **Supplementary Table 3**). These findings indicated that the LSD gene family has followed distinct evolutionary trajectories between monocot and dicot, with stronger functional conservation retained among monocot species.

### 3.5 Analysis of *cis*-acting elements of ZmLSD family members

**Figure 5:**
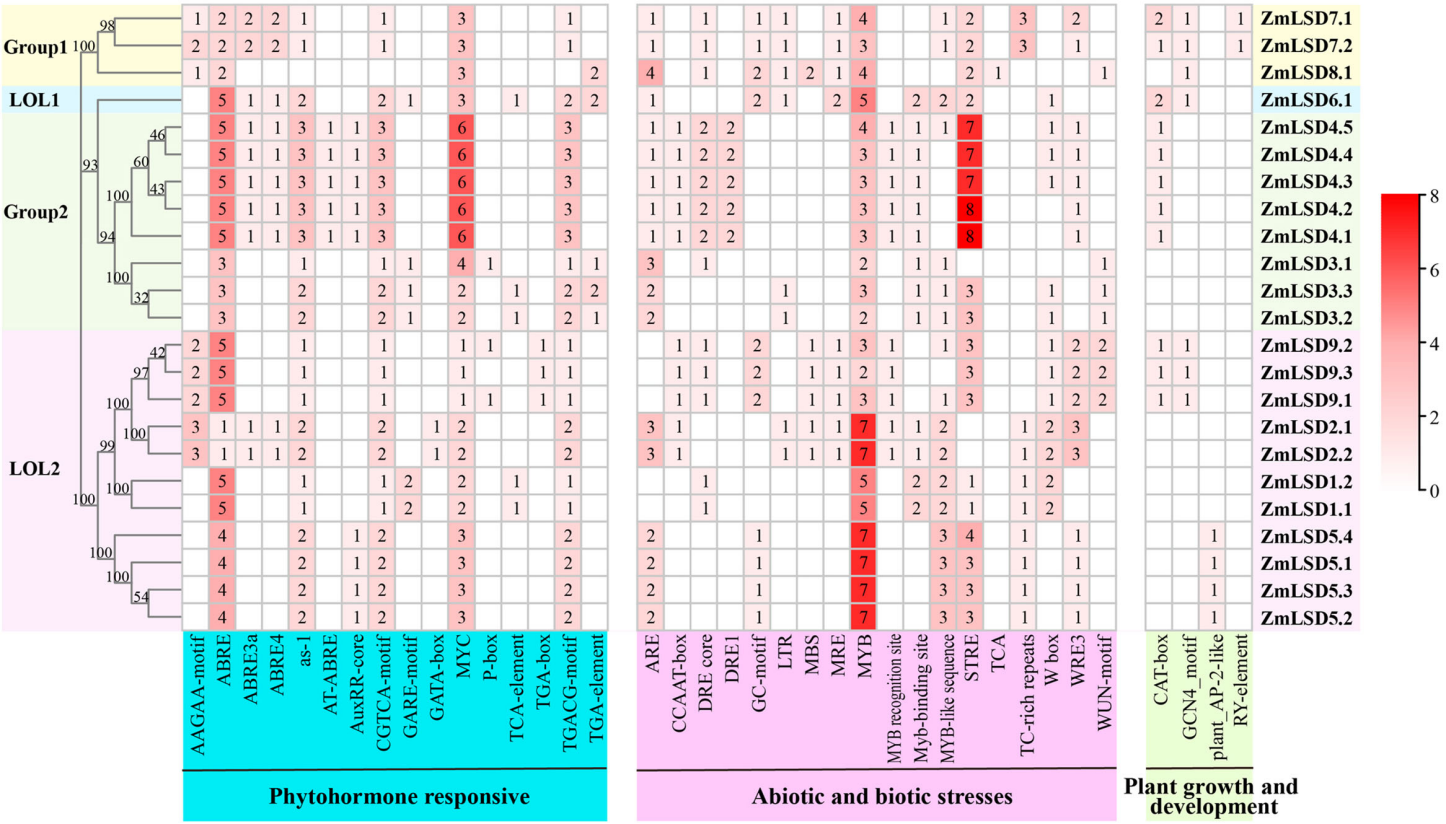
*Cis*-acting elements analysis of *ZmLSDs* promoter. The blue box denotes *cis-*elements associated with plant hormones response, the pink box denotes *cis-*elements related to responses to abiotic and biotic stresses, and the green box denote *cis-*elements associated with plant growth and development.

The promoter sequence of *ZmLSD* genes was extracted by TBtools, and the*cis*-elements were analyzed by PlantCARE. Using Excel, three classes of *cis*-acting elements were screened, which is associated with phytohormone response, abiotic and biotic stresses tolerance, and plant growth and development (**Supplementary Table 4**). Among them, all *ZmLSD* family genes contained ABRE and MYC hormone-responsive elements, and all *ZmLSDs* except for *ZmLSD8*, contained as-1, CGTCA-motif, and TGACG-motif elements (**Figure 5**). Among the biotic and abiotic stress-responsive elements, all *ZmLSD* genes contained MYB elements, and more than 70% of *ZmLSD* family members contain ARE, MYB-like sequence, and STRE *cis*-elements (**Figure 5**). The promoter of *ZmLSDs* also contain *cis*-elements associated with plant growth and development responses. For instance, *ZmLSD7*, *ZmLSD6*, *ZmLSD4*, and *ZmLSD9* contain the CAT-box, expressed in meristematic tissues. The promoter of *ZmLSD7*, *ZmLSD8*, *ZmLSD6*, and *ZmLSD9* contain the GCN4_motif, expressed in the endosperm. The *ZmLSD5* promoter has a plant_AP-2-like element expressed in the seed. The *ZmLSD7* promoter contains an RY-element. These results suggested that LSD genes may participate in regulating plant growth and development, and stress response.

### 3.6 Expression profiles of *ZmLSDs* in different tissues

**Figure 6:**
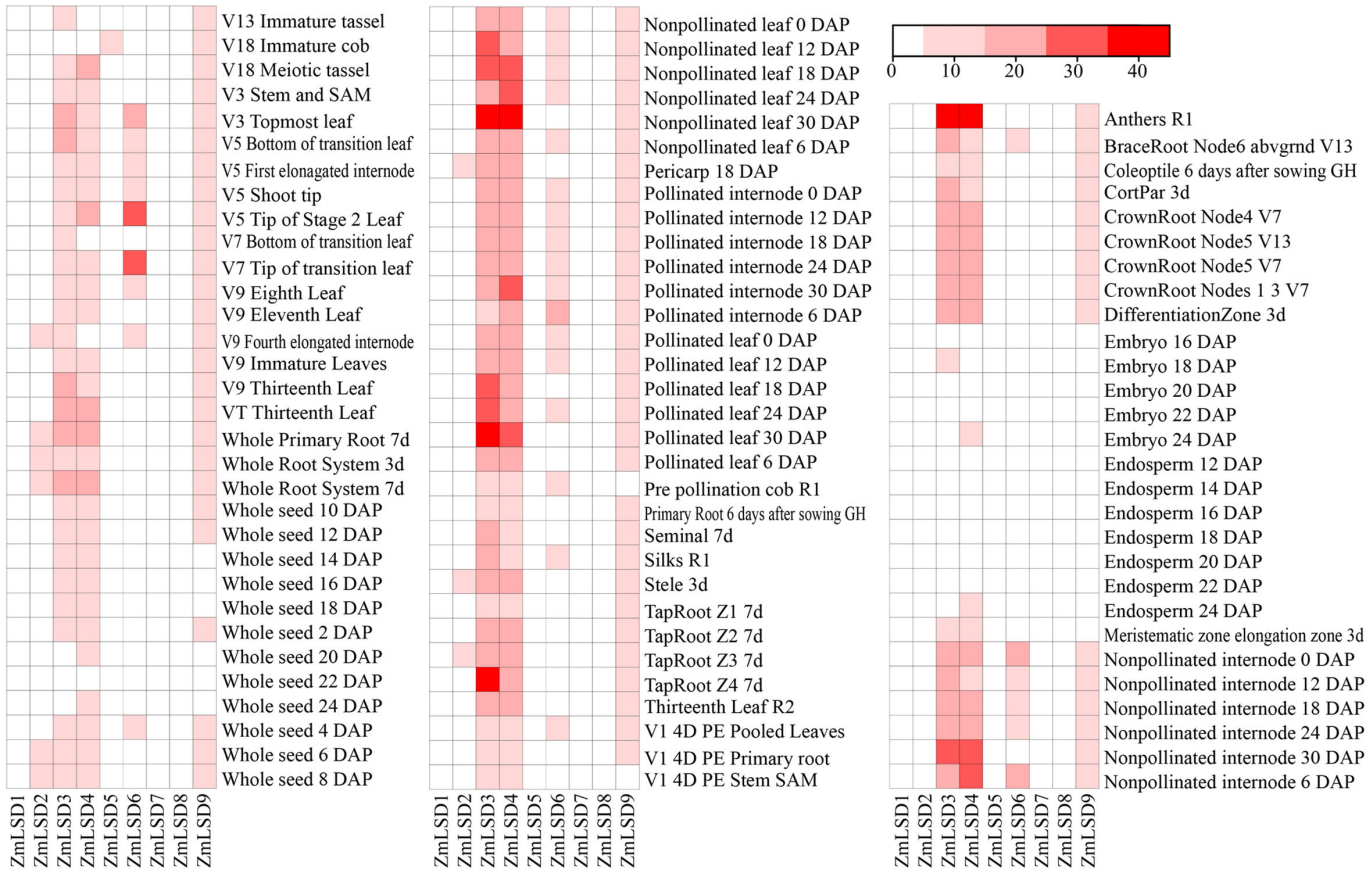
The expression profiles of *ZmLSDs* in various tissues during maize growth and development stages.

To investigate the expression patterns of the *ZmLSD*s in different tissues duringmaize development, we analyzed genes’ fragments per kb exon model per million mapped fragments (FPKM) values using previously reported RNA-seq data (**Supplementary Table 5**). The results showed that among all tissues, only embryos and endosperm exhibit lower expression levels of *ZmLSDs* (**Figure 6**). The expression of *ZmLSD* genes in other parts remained relatively stable throughout all stages of growth and development. The expression levels of *ZmLSD3*, *ZmLSD4*, and *ZmLSD9* were relatively high in all detected tissues, while *ZmLSD1*, *ZmLSD7* and *ZmLSD8* were relatively lower. The *ZmLSD6* expression level was high in some tissues, such as internode, nonpollinated leaf. The above results indicate that the expression of *ZmLSDs* exhibit significant differences and specificity in tissue, suggesting that this family may possess diverse functions during maize growth and development.

### 3.7 Expression patterns of *ZmLSDs* under drought, salt, heat, and ABA hormone stresses

**Figure 7:**
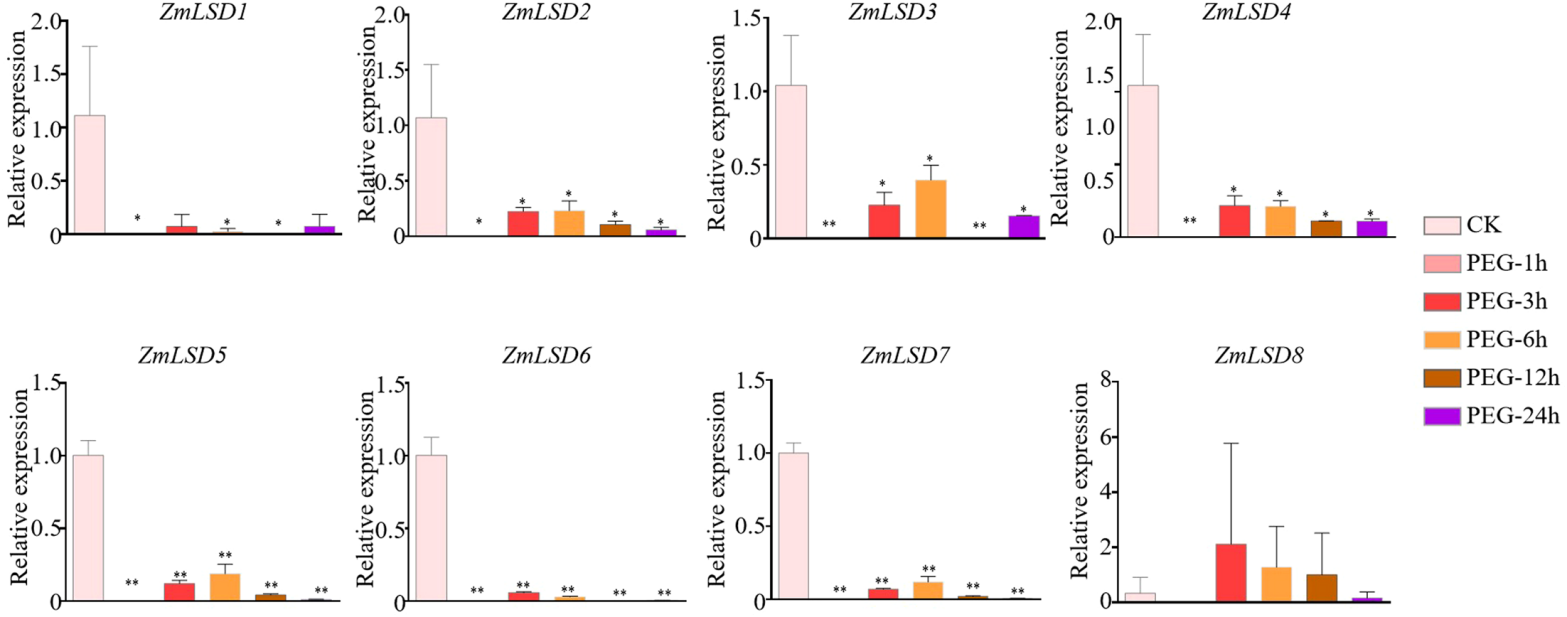
The expression levels of *ZmLSDs* after PEG-6000 treatment. Seedlings with no treatment (PEG-0 h) served as control (CK). The bars indicate the mean ± SD of three replicates. The X-axis indicated different time points after PEG-6000 watered. The Y-axis indicated relative expression. *ZmActin 1* was used as reference gene. * indicates *p* < 0.05; ** indicates *p* < 0.01 (Student’s *t*-test).

**Figure 8:**
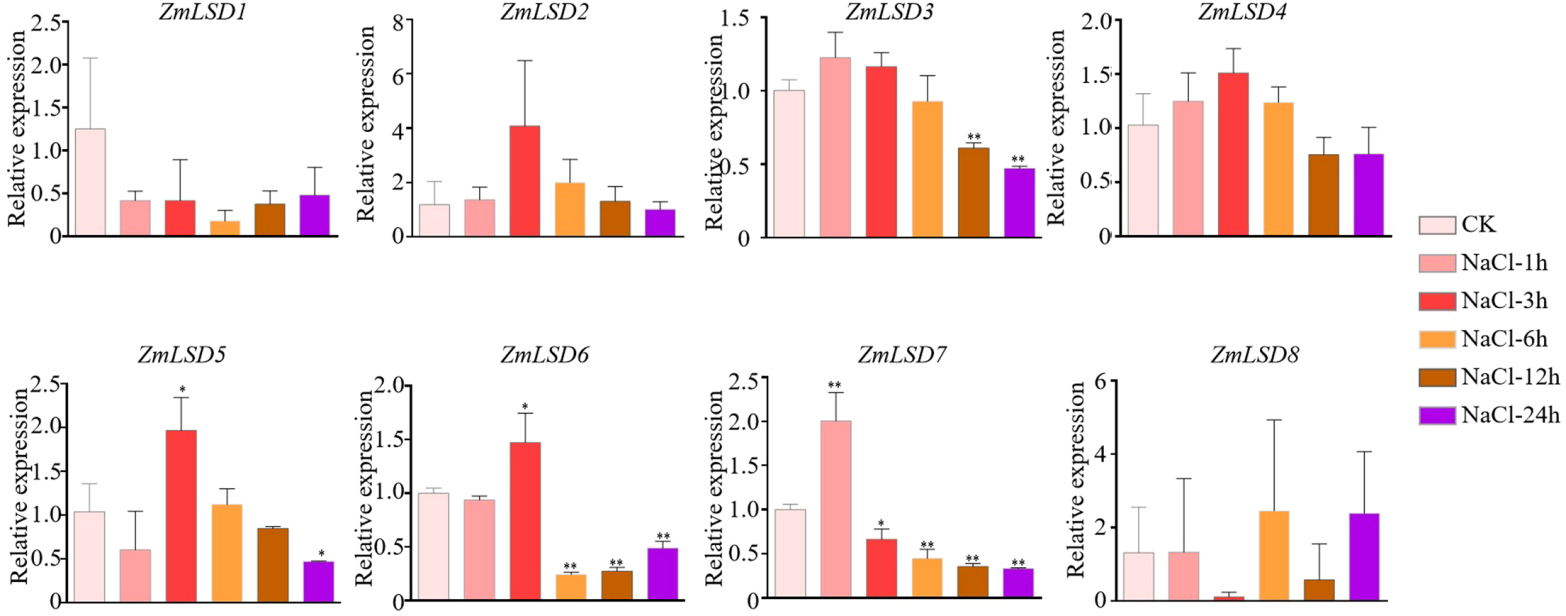
The expression levels of *ZmLSDs* after 200 mM NaCl treatment. Seedlings with no treatment (NaCl-0 h) regard as control (CK). The bars indicate the mean ± SD of three replicates. The X-axis indicated different time points after NaCl watered. The Y-axis indicated relative expression. *ZmActin 1* was used as reference gene. * indicates *p* < 0.05; ** indicates *p* < 0.01 (Student’s *t*-test).

**Figure 9:**
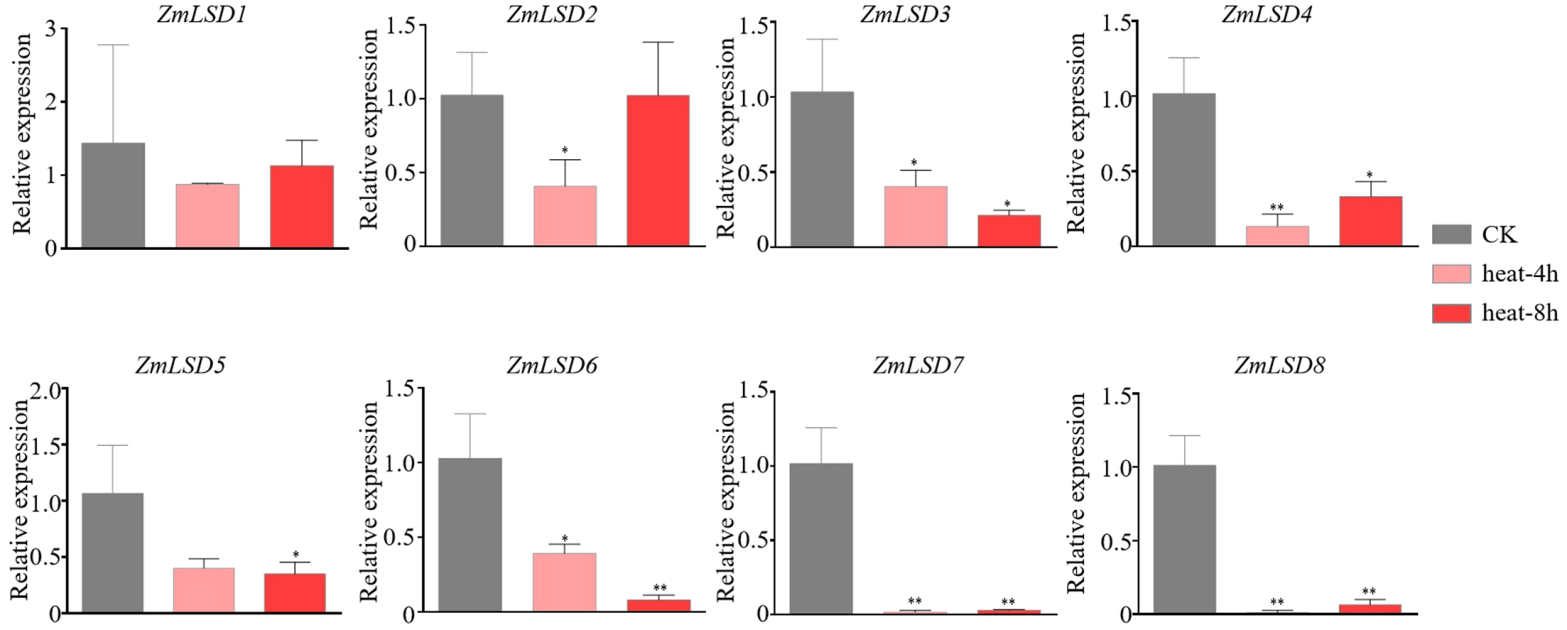
The expression levels of *ZmLSDs* after 42°C heat treatment. Seedlings with no treatment (heat-0 h) served as control (CK). The bars indicate the mean ± SD of three replicates. The X-axis indicated different time points of treatment. The Y-axis indicated relative expression. *ZmActin 1* was used as reference gene. * indicates *p* < 0.05; ** indicates *p* < 0.01 (Student’s *t*-test).

**Figure 10:**
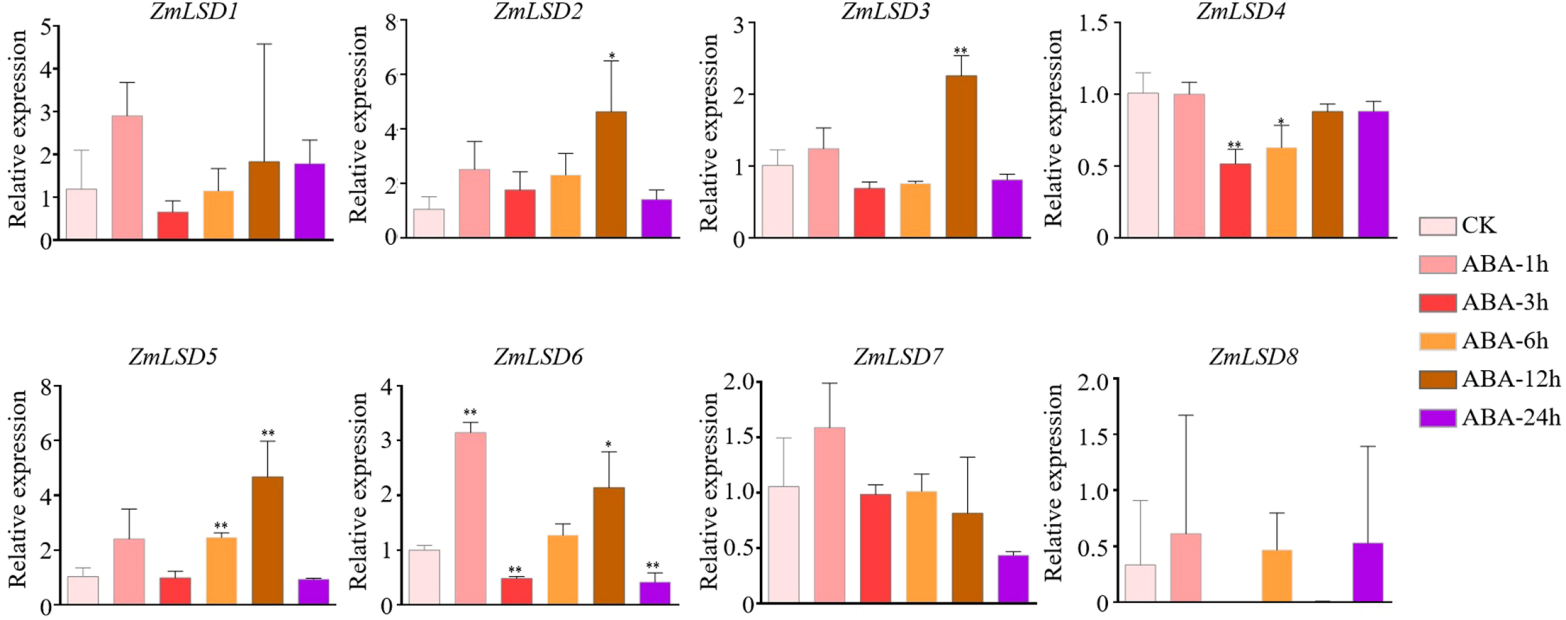
Expression profile of *ZmLSDs* after 100 μM ABA hormone treatment. Seedlings with no treatment (ABA-0 h) regard as control (CK). The X-axis indicated different time points after ABA spray. The Y-axis indicated relative expression. *ZmActin 1* was used as reference gene. The mean values ± SD of three independent experiments are shown. The asterisks indicate significant differences between the control and a subsequent timepoint (Student’s *t*-test). * indicate *p* < 0.05; ** indicate *p* < 0.01.

Previous studies have demonstrated that the LSD family members can participate in responses tobiotic and abiotic stress (Guan et al., 2016; Jiang et al., 2019; Bernacki et al., 2021; Chao et al., 2024). To investigate the expression patterns of *ZmLSD* genes under drought, salt, heat, and hormone ABA stresses, we employed qRT-PCR to detect genes’ expression levels after PEG-6000, NaCl, high temperature, and ABA treatments, respectively (**Supplementary Table 6**). The results showed that after PEG-6000 simulated drought treatment, the expression of genes *ZmLSD2*, *ZmLSD3*, *ZmLSD4*, *ZmLSD5*, *ZmLSD6*, and *ZmLSD7* were significantly down regulated at all time points except for *ZmLSD8* and *ZmLSD1* (**Figure 7**). The expression levels of *ZmLSD1* were significantly downregulated at 1h, 6h, and 9h, after drought stress treatment (**Figure 7**). These findings indicated that *ZmLSDs* participate in maize’s response to drought stress pathway. After salt stress treatment, the expression levels of *ZmLSDs* exhibited an overall pattern of initial increase followed by decline (**Figure 8**). The expression levels of *ZmLSD3* markedly decreased at 12h and 24h after NaCl treatment (**Figure 8**). The expression levels of *ZmLSD5* and *ZmLSD6* showed significant upregulation at 3h post-NaCl treatment, while *ZmLSD7* exhibited marked upregulation at 1h post-treatment; *ZmLSD6* and *ZmLSD7* then exhibited significant downregulation at 6h, 12h, and 24h post-NaCl treatment (**Figure 8**). These results indicated that prolonged salt stress suppresses the expression of ZmLSD family genes. After 4h and 8h of heat treatments, expression levels of all *ZmLSDs* showed a downregulation trend, with the exception of *ZmLSD2*, whose expression at 8h remained largely unchanged compared to the control. Notably, *ZmLSD3*, *ZmLSD4*, *ZmLSD6*, *ZmLSD7*, and *ZmLSD8* exhibited significantly reduced expression levels (**Figure 9**). These findings indicated that ZmLSD family genes participate in maize’s response to heat stress. After ABA hormone treatment at different time points, the relative expression levels of most *ZmLSDs* exhibited a pattern of initial increase, followed by decrease, then rise, and subsequent decline (**Figure 10**). The expression levels of *ZmLSD6* exhibited most robust ABA responsiveness. Compared to the control, the expression levels of *ZmLSD6* showed significant changes at 1h, 3h, 12h, and 24h post-treatment. Notably, its expression level was more than threefold increased after the 1h treatment. In addition, *ZmLSD2*, *ZmLSD3*, and *ZmLSD5* exhibited significant upregulation compared to the control at 12 hours, while *ZmLSD4* showed marked downregulation at 3h after ABA treatment (**Figure 10**). These findings indicated that *ZmLSDs* participate in stress responses to drought, high temperature, salt stress, and the hormone ABA. When subjected to stress, some *ZmLSDs* exhibit consistent expression profile, suggesting that their functions may be redundant.

### 3.8 Subcellular localization of ZmLSD3 and ZmLSD4

**Figure 11:**
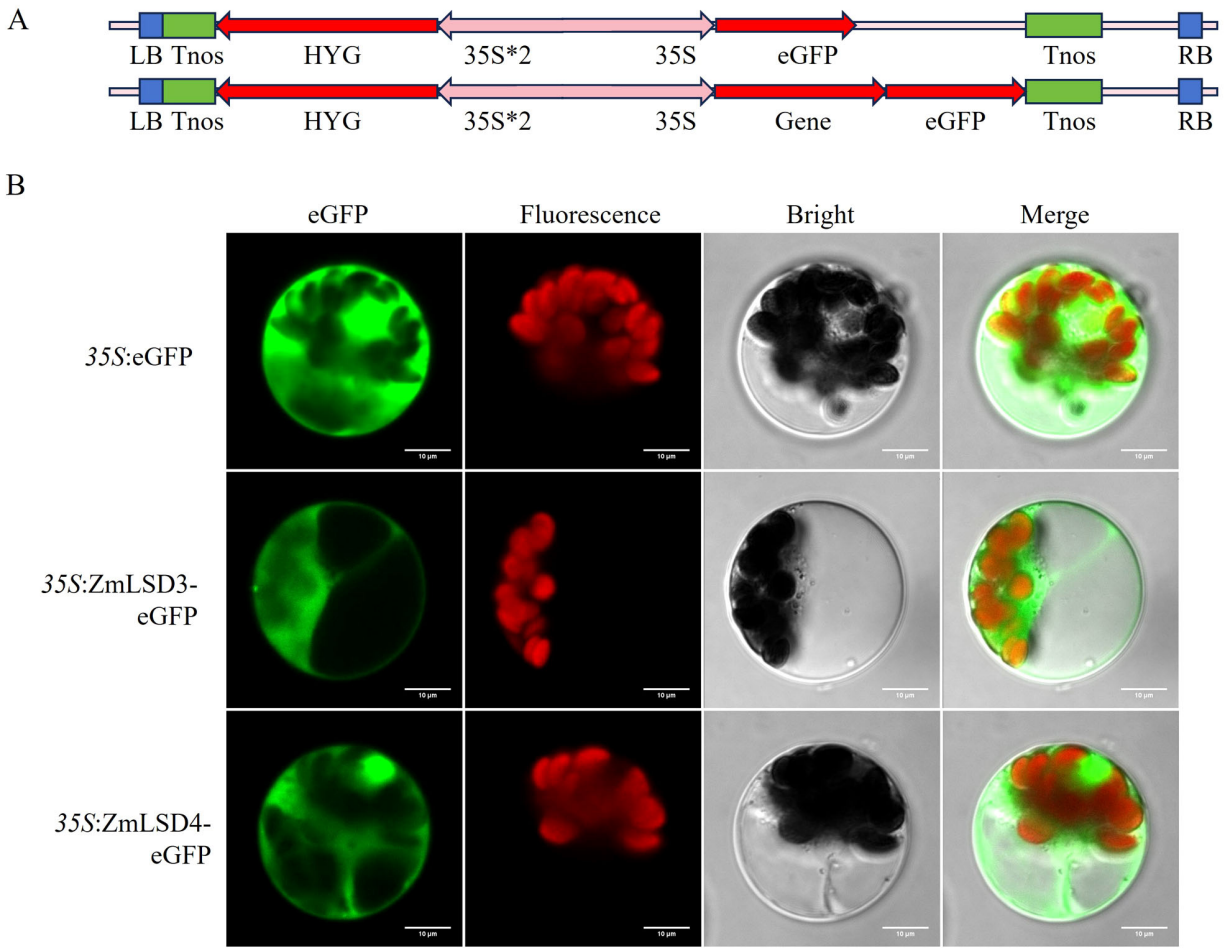
Subcellular localization analysis of ZmLSD3, and ZmLSD4 in maize protoplasts. **(A)** Vector diagram of 35S::eGFP, 35S::ZmLSD-eGFP. **(B)** Fusion proteins were transiently expressed in maize protoplasts. 35S::eGFP vector was used as control, and ZmLSD3 and ZmLSD4, fused with eGFP (green fluorescence). Red fluorescence belongs to chlorophyll in chloroplasts. Bars = 10 μm. Images are representative of three independent experiments.

Determining the subcellular localization of proteins is crucial for studying gene’s function. The PSORT website predicts that most ZmLSDs are localized in the nucleus, cytoplasm, and chloroplast (**Table 1**). To further confirm the localization of this protein family, we selected ZmLSD3 and ZmLSD4, the expression of which showed significantly changed under drought, high temperature, salt stress, and hormonal stress. After designed specific primers (**Supplementary Table 1**), they were cloned from B73 maize using molecular biology techniques. We then constructed *35S::ZmLSD3-eGFP* and *35S::ZmLSD4-eGFP* expression vectors (**Figure 11A**). Following transient transformation of maize protoplasts, subcellular localization of the fusion proteins was examined using laser confocal microscopy. The results revealed that ZmLSD3-eGFP predominantly localized to the cytoplasm, while ZmLSD4-eGFP localized to both the nucleus and cytoplasm (**Figure 11B**). These findings indicated that ZmLSD family members may exert significant functions not only in the nucleus and chloroplasts, but also within the cytoplasm.

## 4 Discussion

Abiotic stress such as drought, salinity, and temperature severely impairs crops growth and development by disrupting physiological processes and upsetting cellular metabolic equilibrium, constituting primary constraints to global agricultural productivity (Zhao et al., 2025). In recent years, researchers have increasingly focused on identifying functional genes that enhance plant stress tolerance. Among the key regulatory elements identified, the LSD transcription factors or scaffold protein has emerged as an important player in orchestrating plant growth regulation, developmental transitions, and adaptive responses to abiotic stress. These DNA-binding proteins exhibit remarkable versatility in modulating gene expression networks associated with cellular defense mechanisms, redox homeostasis maintenance, and programmed cell death regulation (Czarnocka et al., 2017). Recent research highlights their indispensable role in fine-tuning plant adaptation strategies to diverse abiotic challenges, positioning them as promising targets for developing stress-tolerant crop varieties (Akbar et al., 2024; Chao et al., 2024). The LSD gene family has been identified and investigated across numerous species (Cabreira et al., 2013, 2015; Zeng et al., 2022; Chao et al., 2024). However, a comprehensive identification and expression profiling analysis of LSD genes under abiotic stress conditions in maize have not yet been conducted. This research conducted a systematic identification, and analysis of the phylogeny, evolution, expression profiles, and subcellular localization of LSD family genes in maize.

Phylogenetic analysis serves as a fundamental tool for deciphering the evolutionary trajectories of gene families and predicting functional attributes of newly identified genes (Kapli et al., 2020). The phylogenetic relationships reveals that the 23 maize ZmLSD family members and 32 LSDs from three other plant species were classified into five distinct subfamilies (**Figure 1**). Notably, Groups 1 and 2 exhibit exclusive membership from monocotyledonous species, whereas Groups LOL1 and LOL2 include members from both monocotyledons and dicotyledons. Comparative genomic analysis of collinearity patterns provides critical insights into evolutionary divergence. *ZmLSDs* showed no syntenic relationships with the dicotyledonous model organism *Arabidopsis thaliana*, while they display conserved collinear gene pairs with the monocotyledonous species *Sorghum bicolor* and *Oryza sativa* subsp. japonica (**Figure 4**). This phylogenetic distribution pattern strongly suggests that the expansion of the LSD gene family in monocots followed evolutionary pathways fundamentally distinct from those in dicots. Most ZmLSD family members share collinear gene pairs with other monocots, with the exception of *ZmLSD1* (**Figure 3**). This unique evolutionary trajectory of *ZmLSD1* may reflect either accelerated evolutionary rates or functional specialization. Supporting this hypothesis, previous studies have demonstrated that *ZmLSD1* plays a positive role in enhancing the salt tolerance of maize through binding to the *ZmWRKY29* promoter and promoting its expression (Li et al., 2024).

The tissue-specific expression patterns of ZmLSDs provide new insights into their functional roles. *ZmLSDs* expressed in all tissues except embryo and endosperm suggests that *ZmLSDs* may play a significant role throughout maize growth and development (**Figure 6**). This broad expression profile aligns with the functional conservation of LSD genes in other species, where they often act as pivotal regulators of growth-defense trade-offs. *GmLSD* genes exhibits organ-specific distribution within tissues (Cabreira et al., 2013). *OsLOL2* is involved in rice growth through a change in the level of endogenous hormone GA (Xu and He, 2007), and *BohLOL1* participates in bamboo growth regulation by integrating environmental cues with hormonal signaling pathways (Yeh et al., 2011). Thus, we infer that *ZmLSDs* also participate in maize plant growth and development, particularly the genes *ZmLSD3*, *ZmLSD4*, *ZmLSD6*, and *ZmLSD9*, which exhibit higher expression levels across various developmental stages.

*Cis*-elements serve as critical regulatory in signaling pathways involved in transcriptional regulation. The promoter regions of *ZmLSDs* are rich in *cis*-elements involved in biotic and abiotic stress, plant hormones, and growth and development (**Figure 5**). The ABA-dependent pathway is one of the primary pathways involved in drought stress responses. All *ZmLSDs* promoter region contain ABRE, MYC, and MYB *cis*-elements. Under drought stress, the expression level of all *ZmLSD*s downregulated, indicating that *ZmLSDs* may be involved in drought stress responses (**Figure 7**). *PagLOL1b* improves drought tolerance by modulating stomatal closure and ROS scavenging, demonstrating the functional relevance of LSD genes in water deficit adaptation (Chao et al., 2024). The observed downregulation of *ZmLSDs* under drought implies maize may help itself survive drought stress by reducing the mRNA expression level of *ZmLSDs*. Furthermore, the expression levels of this family genes exhibited dynamic expression patterns under heat and salt stress, as well as ABA treatment (**Figures 8**-**10**), highlighting their integration into multiple stress signaling networks. Under heat stress,*ZmLSD4* showed significant downregulation after 4 h and 8 h treatments, which is likely related to the presence of abundant STRE *cis*-elements in its promoter. After ABA treatment, the expression levels of *ZmLSD4*, *ZmLSD5*, and *ZmLSD6* showed significant changes, which is consistent with the fact that its promoter contains abundant ABRE, MYC, and MYB *cis*-elements. Additionally, the upstream regions of *ZmLSDs* promoter contain MYC and as-1 elements implicate these genes involved in jasmonic acid and salicylic acid mediated defense responses. This crosstalk between hormone signaling pathways suggests that *ZmLSDs* act as molecular hubs to balance growth and survival under stress. Besides, the *AtLSD* counterbalances the formation of aerobic tissues under hypoxia conditions by negatively regulating lysigenous aerenchyma formation (Muhlenbock et al., 2007). In maize, anaerobic response *cis*-elements (ARE) are present in the promoters of 78% of *ZmLSD* genes (**Supplementary Table 4**), suggesting that the *ZmLSD* gene family may also participate in anaerobic responses. Moreover, LSD transcription factors can bind to the *SUT1-T1* promoter in *S. officinarum* and negatively regulate *SUT1-T1* in *Erianthus rufipilus and Saccharum officinarum*, which plays a pivotal role in sugar transport (Akbar et al., 2024). The *MeLSD3* could regulates oxidative stress response via fine-tuning *MeAPX2* activity (Zeng et al., 2022) and regulates bacterial blight resistance through fine-tuning *MeSRT1* histone acetylation in cassava (Zeng et al., 2023). MeLSD3 is localized in the cytoplasm and the nucleus, the same localization as ZmLSD4. Therefore, we infer that theZmLSD4 may function in both the nucleus and the cytoplasm. Future research should employ genetic transformation experiments (overexpression or loss-of-function lines) to directly demonstrate the specific function of the ZmLSD genes in abiotic stress resistance and integrate multi-omics data to elucidate the functional diversification mechanisms and their potential applications in agriculture.

## 5 Conclusion

We provide a comprehensive genomic identification and expression profile analysis of the LSD gene family in maize. There are nine *ZmLSDs* encoding 23 proteins with diverse physicochemical properties, distributed across five chromosomes. Phylogenetic analysis classified these genes into four distinct subfamilies, revealing evolutionary conservation through syntenic relationships with sorghum and rice homologs. *ZmLSDs* exhibit tissue-specific expression patterns, with *ZmLSD3*, *ZmLSD4*, and *ZmLSD9* showing particularly broad expression profiles. Crucially, our data suggest that *ZmLSDs* may be involved in abiotic stress responses, particularly under drought and heat stress, where most members were significantly downregulated. The differential subcellular localization of ZmLSD3 and ZmLSD4 suggests that ZmLSD3 may mainly function in the cytoplasm, while ZmLSD4 may play a role in both the nucleus and the cytoplasm. These results establish a crucial foundation for understanding the expression profile of *ZmLSDs* in responses to environmental challenges, offering potential targets for improving stress resilience in breeding programs.

## Data Availability

The original contributions presented in the study are included in the article/**Supplementary Material**. Further inquiries can be directed to the corresponding author.
