# Supplementary figures and images for: Identification and expression profiling of LSD genes reveal their role in developmental and abiotic stress conditions in maize
Source: Front Plant Sci. 2026 Feb 10;17:1760884. doi: 10.3389/fpls.2026.1760884 (PMC12929109; doi:10.3389/fpls.2026.1760884)

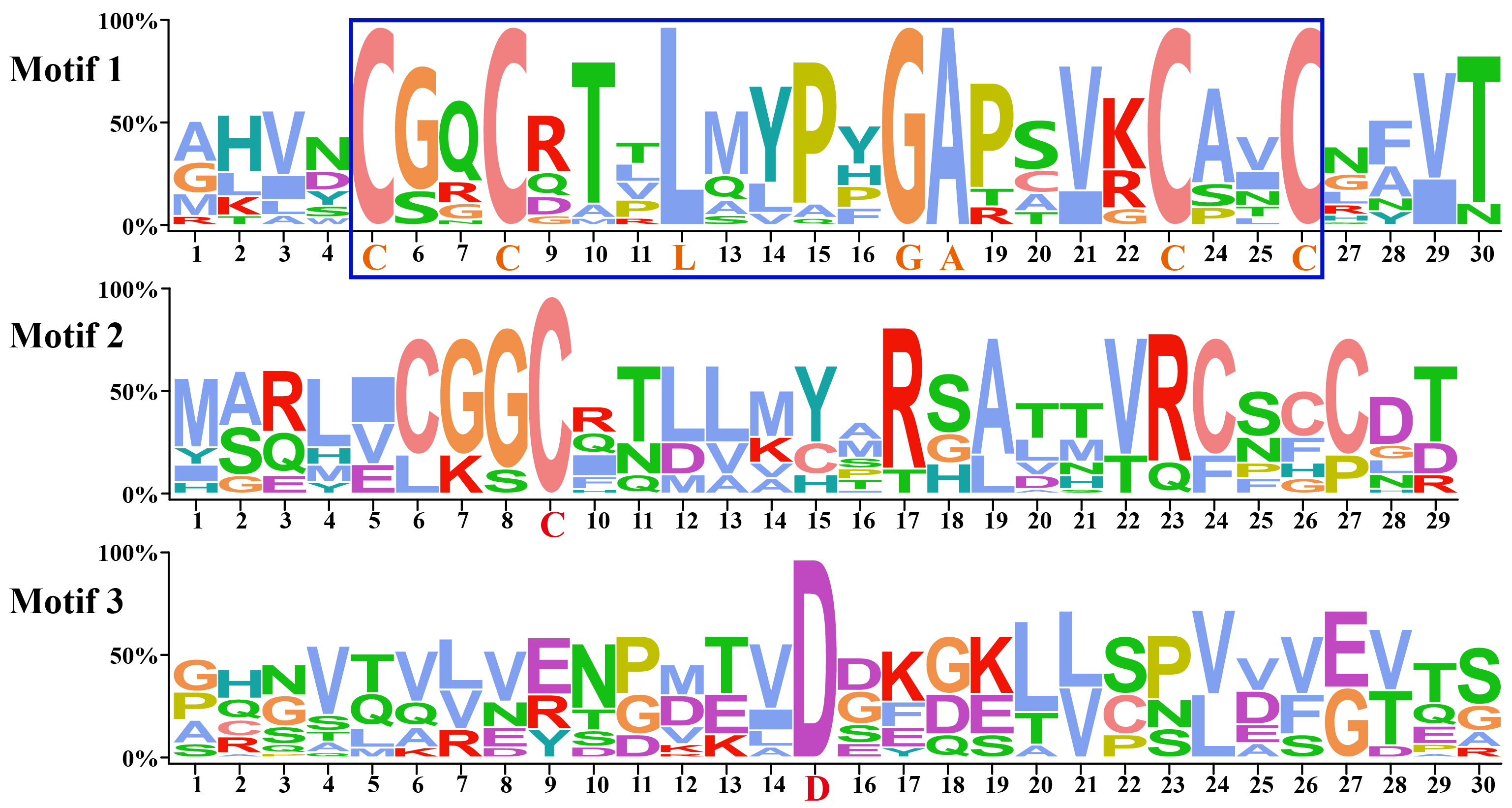

Supplement: Supplementary file 1 [file Image1.jpeg]
